# The Interaction of *TPH1* A779C Polymorphism and Maternal Authoritarianism on Creative Potential

**DOI:** 10.3389/fpsyg.2018.02106

**Published:** 2018-11-02

**Authors:** Jinghuan Zhang, Xiao Han, Si Si, Shun Zhang

**Affiliations:** Department of Psychology, Shandong Normal University, Jinan, China

**Keywords:** creativity, creative potential, *TPH1*, maternal authoritarianism, gene-environment interaction

## Abstract

Exploring the possible mechanisms through which gene may interact with environment to influence creativity has been one of the leading issues in creativity research. In a sample of four hundred and twenty-one Chinese undergraduate students, the present study investigated for the first time the interaction of *TPH1* A779C polymorphism and maternal parenting styles on creative potential. The results showed that there was a significant interaction of *TPH1* A779C polymorphism and maternal authoritarianism on creative potential. Moreover, the analysis of regions of significance (Ros) provided supporting evidences for both the diathesis-stress model (flexibility) and the differential susceptibility model (originality). These findings extend our understanding concerning the mechanisms by which gene and environment may act in coordination to contribute to creativity.

## Introduction

Recent developments in molecular genetics have inspired a number of studies to explore the genetic correlates of creativity and to identify genes associated with creativity. Among the candidate genes, the most extensively studied is the tryptophan hydroxylase 1 gene (*TPH1*). *TPH1* is located on chromosome 11, and is expressed in both human central and peripheral nervous system (Zill et al., 2007). The enzyme encoded by this gene is the rate-limiting enzyme in the biosynthesis of serotonin, and regulates serotonin levels by converting tryptophan to 5-hydroxytryptophan which is the direct precursor of serotonin (Hamon et al., 1981).

Previous studies examining the association of *TPH1* and creative potential generally focused on the role of *TPH1* A779C polymorphism (rs1799913), but yielded inconsistent results (Reuter et al., 2006; Runco et al., 2011; Zhang and Zhang, 2017). Reuter et al. (2006) first examined the association of *TPH1* A779C polymorphism and creative potential, and the results indicated that this polymorphism was associated with total creative potential score. Based on Reuter et al.’s work, Runco et al. (2011) further investigated the association of *TPH1* A779C polymorphism and the three core dimensions (fluency, flexibility, and originality) of creative potential, and demonstrated that *TPH1* A779C polymorphism was only associated with fluency. By including both tag single nucleotide polymorphisms (SNPs) and functional SNPs, Zhang and Zhang (2017) recently for the first time systematically explored the association of *TPH1* and creative potential, but found that *TPH1* A779C polymorphism was not related to any of the three core dimensions of creative potential.

As for these inconsistent results, there are a number of possible reasons (e.g., sample size, age, and gender). However, since creativity, like most of other complex traits, is determined by the interplay of gene and environment, it is reasonable to suspect that the primary reason that accounts for the discrepancy may be attributed to the neglect of gene–environment (G × E) interaction (Rutter et al., 2006).

One possible environmental factor that may interact with *TPH1* A779C polymorphism to influence creative potential is maternal parenting styles. Parenting styles represent the emotional connections and the quality of contacts parents make with their children, and it has been shown that maternal parenting styles are critical environmental factors for individual’s creativity (Nichols, 1964; Lim and Smith, 2008). Existing studies regarding the relationship between maternal parenting styles and creativity have yielded inconsistent results (Miller et al., 2012; Fearon et al., 2013; Mehrinejad et al., 2015), while a recent study provided a new perspective to re-evaluate the relationship by showing that *DRD2* genotype (rs1799732) could interact with maternal parenting styles to affect creative potential (Si et al., 2018). This finding suggested that the influence of maternal parenting styles on creativity may depend on the genotypes of specific genes, and the true effect of relevant genes on creativity may not be detected unless the target sample is stratified by environmental factors (e.g., maternal parenting styles). Thus, to test whether the discrepancy regarding the effect of *TPH1* A779C polymorphism on creativity is caused by the neglect of potential G × E interaction, the present study was designed to examine the interaction of *TPH1* A779C polymorphism and maternal parenting styles on creative potential. It is hypothesized that creative potential of individuals carrying different genotypes of *TPH1* A779C polymorphism may be differently affected by maternal parenting styles.

To interpret the mechanisms by which gene may interact with environment, two overarching theoretical perspectives have been proposed: the diathesis-stress model and the differential susceptibility model. The diathesis-stress model largely focuses on the negative environments and suggests that only individuals with the “risk” alleles are more prone to be affected by negative environments (Belsky, 1997; Caspi and Moffitt, 2006). In contrast, the differential susceptibility model focuses on both the positive and the negative environments, and suggests that genes could be “plasticity” rather than “risk.” Individuals with the “plasticity” alleles are not only adversely affected by negative environments, but also benefit the most from positive environments (Belsky and Pluess, 2009; Ellis et al., 2011). Among the two perspectives, the diathesis-stress model is most commonly employed, and most of the extant studies regarding G × E interaction have been conducted under this framework (Monroe and Simons, 1991; Burmeister et al., 2008). However, there has also been a rapid growing body of supporting evidence has highlighted the importance of the differential susceptibility model. For example, a recently published meta-analysis showed that many studies, especially in the last 5 years, have found supporting evidence for the differential susceptibility model (Slagt et al., 2016).

As for creativity, to the best of the authors’ knowledge, there has been only one study that has examined G × E interaction. Although the study found a significant interaction of *DRD2* and parenting styles on creative potential (Si et al., 2018), whether the finding would be consistent with the diathesis-stress model or the differential susceptibility model was not systematically tested. Thus, until now, little is known about the exact G × E interaction pattern for creativity. To further clarify the G × E interaction pattern for creativity as well as to better explain the potential interaction of *TPH1* A779C polymorphism and maternal parenting styles, the present study also examined whether the potential gene × parenting interaction would coincide with the diathesis-stress model or the differential susceptibility model.

## Materials and Methods

### Participants

The participants were four hundred and twenty-one unrelated healthy Han Chinese undergraduate students from Shandong Normal University (100 males and 321 females, mean age = 18.92 years old). The present study was approved by the Institutional Review Board of Shandong Normal University. Written informed consent was obtained from each participant.

### Measures

#### Creative Potential

Creative potential was assessed by three Uses tasks selected from Runco’s Creativity Assessment Battery (rCAB). The Uses tasks asked the participants to list as many as possible uses for three common subjects (toothbrush, tire, and spoon), and were comparable to other assessments of creative potential (Wallach and Kogan, 1965; Guilford, 1968). For each task, four-minute was allowed. The tasks were scored for the three core dimensions of creative potential (fluency, flexibility, and originality). Fluency score was the total number of responses given by the participant. Flexibility score was the number of different categories of the participant’s responses. Originality score was the number of unusual responses (given by less than 5% of the sample). Two trained raters scored all three tasks. The inter-rater reliabilities for all three scores were higher than 0.95.

#### Maternal Parenting Styles

Maternal parenting styles were self-reported by participants using the Parental Authority Questionnaire (PAQ) which was developed to measure the authoritative, authoritarian and permissive parental authority prototypes proposed by Baumrind (1971). It consisted of 30 items rated on a 5-point scale ranging from 1 (weakly disagree) to 5 (strongly agree). The Cronbach’s alpha for maternal authoritative parenting, maternal authoritarian parenting and maternal permissive parenting was 0.75, 0.77, and 0.64, respectively.

#### Genotyping

Peripheral venous blood samples from each participant were first collected with the assistance of medical staff. Genomic DNA was extracted from peripheral venous blood samples by using the Qiagen QIAamp DNA Mini Kit. Genotyping for *TPH1* A779C polymorphism were performed at Beijing Genomics Institute-Shenzhen by using the Sequenom MassARRAY iPLEX system according to the manufacturer’s instructions. For quality control, 5% random DNA samples were genotyped twice to calculate genotyping error. The genotyping accuracy was 100%.

#### Statistical Analysis

To test the interaction of *TPH1* A779C polymorphism and maternal parenting styles on creative potential, hierarchical multiple linear regression was conducted by using SPSS version 24.0. The procedure was as follows: covariates (gender) were entered in the first step to control for the confounding effects. The main effect of *TPH1* A779C polymorphism (dummy-coded as 0 = CC versus 1 = AA/AC) and maternal parenting styles were entered in the second step. Finally, the interaction terms for *TPH1* A779C polymorphism and maternal parenting styles were added in the third step. When significant interaction was found, simple slope analysis was conducted by using the PROCESS macro for SPSS version 2.16 (Hayes, 2013).

To test whether the significant G × E interaction supported the diathesis-stress or the differential susceptibility model, the analysis of regions of significance (RoS) recommended by Roisman et al. (2012) was carried out by using a web-based program^1^. Briefly, this analysis includes three indexes and a test for non-linearity. First, the RoS on *X* index (Ros *X*) represents the upper and lower bounds of values for the predictor (*X*) at which the regression of the outcome (*Y*) on the moderator (*Z*) is statistically significant. If the association between the moderator (*Z*) and the outcome (*Y*) is significant at both the upper and lower ends of the predictor (*X*) within +/−2 *SD*, then the differential susceptibility model is supported. Second, the proportion of interaction index (PoI) measures the proportion of the total interaction that is represented on the right side or the left side of the crossover point for the interaction, indicating how much a crossover interaction is “for better” or “for worse.” PoI values between around 0.40 and 0.60 indicate an interaction highly consistent with the differential susceptibility model, while PoI values close to 0 or 1 suggest strong supporting evidence for the diathesis–stress model. Third, the proportion affected index (PA) estimates the proportion of the population that is differentially affected by the moderator (*Z*). PA values around 0.50 indicate strong evidence for the differential susceptibility model, while PA values close to 0 or 1 provide strong evidence for the diathesis-stress model. Finally, the test of non-linear effects ascertains whether the apparent differential susceptibility effect could be artifacts of imposing a linear model on a non-linear diathesis-stress phenomenon. To support the differential susceptibility effect, the results of the model must show that the linear interaction term remains significant after controlling for the non-linear terms (quadratic effects: *X*^2^ and *ZX*^2^).

## Results

### Descriptive Statistics

The number of participants as well as the frequencies for *TPH1* A779C polymorphism genotypes were CC (130, 30.9%), AC (201, 47.7%), and AA (90, 21.4%), respectively. No deviation from Hardy-Weinberg equilibrium was observed (*p* = 0.49). Table 1 shows the descriptive statistics and the correlation matrix. Most notably, maternal authoritativeness was positively correlated with fluency and originality. Neither *TPH1* A779C polymorphism nor other maternal parenting styles was correlated with the three scores of creative potential.

**Table 1:** Descriptive statistics and correlations among the studied variables.

	1	2	3	4	5	6	7	8
1. Gender	1							
2. *TPH1* A779C polymorphism	0.035	1						
3. M-authoritativeness	0.031	−0.013	1					
4. M-authoritarianism	0.072	0.065	−0.248^∗∗^	1				
5. M-permissiveness	−0.038	−0.007	0.222^∗∗^	−0.245^∗∗^	1			
6. Fluency	−0.151^∗∗^	0.057	0.110^∗^	−0.020	0.075	1		
7. Flexibility	−0.052	0.066	0.050	−0.016	0.065	0.789^∗∗^	1	
8. Originality	−0.050	0.015	0.118^∗^	−0.033	0.022	0.845^∗∗^	0.657^∗∗^	1
*M*	–	–	25.43	13.44	17.57	8.41	4.42	2.53
*SD*	–	–	5.48	6.41	4.58	2.92	1.09	1.69

*^∗^p < 0.05, ^∗∗^p < 0.01. M-authoritativeness, maternal authoritativeness; M-authoritarianism, maternal authoritarianism; M-permissiveness, maternal permissiveness; Gender was dummy-coded as 0, female versus 1, male; Genotypes of TPH1 A779C polymorphism were dummy-coded as 0, CC versus 1, AA/AC.*

### Regression Models

**FIGURE 1:**
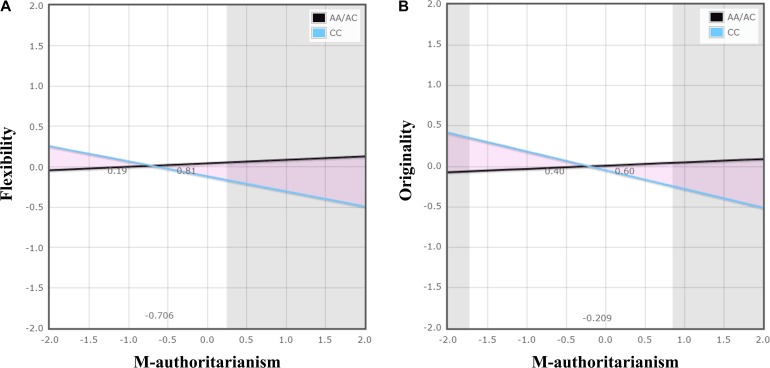
The interaction of *TPH1* A779C polymorphism and maternal authoritarianism on creative potential. The gray shaded areas represent regions of significance (RoS) on *X* and denote where the two lines differ significantly from each other within +/−2 *SD*. The triangular pink shaded areas depict the proportion of the interaction (PoI). Graph **(A)** demonstrates a diathesis-stress effect: the CC genotype differed from the AA/AC genotype, showing significantly lower flexibility when there was high maternal authoritarianism. Graph **(B)** demonstrates a differential susceptibility effect: the CC genotype, with respect to the AA/AC genotype, showing significantly higher originality when there was low maternal authoritarianism, as well as significantly lower originality when there was high maternal authoritarianism. The figure was produced by using a web-based program developed by Chris Fraley http://www.yourpersonality.net/interaction/ to perform the RoS analysis as recommended by Roisman et al. (2012).

Hierarchical multiple linear regression analysis revealed significant *TPH1* A779C polymorphism × maternal authoritarianism interactions on flexibility and originality (Table 2). No other significant interactions was observed (see Supplementary Tables S1–S3). For the significant *TPH1* A779C polymorphism × maternal authoritarianism interactions, simple slopes analysis showed that maternal authoritarianism marginally negatively predicted flexibility for individuals with the CC genotype (β = −0.187, *t* = −1.96, *p* = 0.05), but not for those with the AA/AC genotype (β = 0.043, *t* = 0.757, *p* > 0.05); maternal authoritarianism negatively predicted originality for individuals with the CC genotype (β = −0.232, *t* = −2.43, *p* < 0.05), but not for those with the AA/AC genotype (β = 0.041, *t* = 0.714, *p* > 0.05) (Figures 1A,B).

**Table 2:** Significant results of hierarchical multiple linear regression analysis testing the interaction of *TPH1* A779C polymorphism and maternal authoritarianism on creative potential.

	Flexibility									Originality								
	Model I			Model II			Model III			Model I			Model II			Model III		
	*B (SE)*	β	*t*	*B (SE)*	β	*t*	*B (SE)*	β	*t*	*B (SE)*	β	*t*	*B (SE)*	β	*t*	*B (SE)*	*b*	*t*
***Control variables***																		
Gender	−0.132 (0.125)	−0.052	−1.06	−0.135 (0.125)	−0.053	−1.08	−0.129 (0.125)	−0.051	−1.04	−0.199 (0.194)	−0.050	−1.03	−0.193 (0.195)	−0.048	−0.988	−0.182 (0.194)	−0.046	−0.939
***Independent variables***																		
M-authoritarianism	–	–	–	−0.003 (0.008)	−0.017	−0.345	−0.005 (0.008)	−0.028	−0.575	–	–	–	−0.008 (0.013)	−0.030	−0.620	−0.012 (0.013)	−0.044	−0.894
*TPH1*	–	–	–	0.162 (0.115)	0.069	1.41	0.177 (0.115)	0.075	1.54	–	–	–	0.069 (0.179)	0.019	0.384	0.096 (0.179)	0.026	0.539
M-authoritarianism × *TPH1*	–	–	–	–	–	–	0.039 (0.019)	0.101	2.07^∗^	–	–	–	–	–	–	0.072 (0.029)	0.120	2.46^∗^
***Model***																		
*R*^2^	0.003			0.008			0.018			0.002			0.004			0.018		
Δ*R*^2^	0.003			0.005			0.010			0.002			0.001			0.014		

*^∗^p < 0.05; M-authoritarianism, maternal authoritarianism; Gender was dummy-coded as 0, female versus 1, male; Genotypes of *TPH1* A779C polymorphism were dummy-coded as 0, CC versus 1, AA/AC.*

### Analysis of RoS

For flexibility, the analysis of RoS provided supporting evidence for the diathesis-stress model (Figure 1A and Table 3). The RoS on *X* test revealed that the regression of flexibility on *TPH1* A779C polymorphism was significant at the upper bound of maternal authoritarianism within +2 *SD* of the mean, but the lower bound was less than −2 *SD*. The PoI indexes suggested that less than 20% of the interaction occurred left of the crossover point (because maternal authoritarianism negatively predicted flexibility, the left of the crossover point represents “for better”), whereas over 80% was right of the crossover point (“for worse”). The PA indexes indicated that 24% of individuals were affected “for better” (also because maternal authoritarianism negatively predicted flexibility), while 76% were affected “for worse.”

**Table 3:** Diathesis–stress/differential susceptibility indices for the interaction of *TPH1* and maternal authoritarianism on creative potential.

Creative potential	RoS *X* (M-authoritarianism)		PoI (for better)	PoI (for worse)	Crossover	PA (for better)	PA (for worse)	Test of non-linearity	Best fitting model
	Upper bound	Lower bound							
Flexibility	0.248	−11.92	0.19	0.81	−0.706	0.24	0.76	–	Diathesis–stress
Originality	0.853	−1.73	0.40	0.60	−0.209	0.42	0.58	Passed	Differential susceptibility

*RoS X, regions of significance on X; M-authoritarianism, maternal authoritarianism; PoI, proportion of the interaction index; Crossover denotes the value of X (maternal authoritarianism) at which the genotype group regression lines intersected; PA, proportion affected index.*

For originality, the analysis of RoS demonstrated supporting evidence for the differential susceptibility model (Figure 1B and Table 3). The RoS on *X* test revealed that the regression of originality on *TPH1* A779C polymorphism was significant at both the upper and lower bound of maternal authoritarianism within +/−2 SD of the mean. Both the PoI and the PA indexes were close to 0.50. Test of non-linearity revealed significant quadratic effects of *X*^2^ (maternal authoritarianism × maternal authoritarianism interaction). However, when controlling for the non-linear term by adding it to the original model, the interaction of *TPH1* A779C polymorphism and maternal authoritarianism remained statistically significant.

## Discussion

Previous research focused on the effect of *TPH1* A779C polymorphism and parenting styles on creative potential has both produced mixed results; however, the exact reason concerning the discrepancies has not yet been identified. By revealing the interaction of *TPH1* A779C polymorphism and maternal parenting styles on creative potential, the present study suggested that the neglect of potential G × E interaction might be one of the primary reasons that account for the discrepancies.

In the present study, significant interaction of *TPH1* A779C polymorphism and maternal authoritarianism was found on creative potential. It was shown that the negative effect of maternal authoritarianism on creative potential was only present for individuals with the CC genotype, but not for those with the AA/AC genotype, suggesting that the CC genotype might be more sensitive to high maternal authoritarianism.

The *TPH1* encodes the rate-limiting enzyme for serotonin biosynthesis in the neurons of the raphe nuclei (Nakamura et al., 2006; Zill et al., 2007), and thus regulates serotonin levels and influences behaviors controlled by serotonin. Among *TPH1*-related genetic variants, the most extensively studied is the A779C polymorphism. The A779C polymorphism is located in the intron regions of *TPH1*. Although not directly leading to functional change in protein coding, this polymorphism may affect *TPH1* expression by influencing the binding affinity of GATA-1 transcription factor (Nielsen et al., 1997). And it has been shown that this polymorphism was associated with individual differences in serotonin production and basal serotonin levels. Compared with the AA/AC genotype, the CC genotype was associated with higher serotonin production and higher basal serotonin levels (Jönsson et al., 1997).

Serotonin plays a pivotal role in stress management (Chaouloff et al., 1999; Chaouloff, 2000; Holmes, 2008). Serotonin released in response to psychological stress has been demonstrated to serve a stress-buffering function to attenuate the damaging effect, and thus increases the modulatory capacity of the stress response system (Mitchell et al., 1990; Gesing et al., 2001; Laplante et al., 2002). During childhood period, parents largely control and moderate children’s life and social environment, and are therefore the main sources of stress to children. Children exposed to harsh parenting, such as high maternal authoritarianism, may perceive the environment as stressful and experience higher levels of psychological control and stress (Deater-Deckard, 1998). To mitigate the damaging effect of high maternal parenting stress, a persistent release of serotonin as well as a prolonged activation of serotonin production may be instinctively induced. Obviously, this process could benefit children by protecting them from the damaging effect of psychological stress associated with high maternal authoritarian parenting; however, accompanied by this process, there might also be long term neurobiological influences on brain development and cognitive functions.

Creativity might be among the cognitive functions that being influenced by this process. Previous findings concerning the effect of serotonin on creativity have suggested that serotonin plays an important but complex role in creativity. While lower serotonin levels may harm creativity by decreasing cognitive flexibility, higher serotonin levels may also impair creativity by decreasing approach motivation and avoidance motivation (Nutt et al., 2007; Cools et al., 2008; Flaherty, 2011). This complicated relationship has particularly important implications for understanding the influence of the prolonged serotonin production on creativity: since the overall higher serotonin levels may harm creativity, when considering the effect of the prolonged serotonin release on creativity, individual differences in serotonin production and basal serotonin levels must be taken into account. And this may partially explain why the negative effect of maternal authoritarianism on creative potential was only present for individuals with the CC genotype. As mentioned above, for individuals exposed to high maternal authoritarianism, a prolonged activation of serotonin production may be instinctively induced to mitigate the negative effect of high psychological stress. Besides increasing the modulatory capacity of stress, the elevated serotonin levels may also exert an effect on individuals’ creative potential. Importantly, for individuals who were of higher serotonin production and higher basal serotonin levels (CC genotype), the prolonged activation of serotonin production may result in excessive overall serotonin levels, and thus finally impairs creative potential.

To clarify the G × E interaction pattern for the significant interaction of *TPH1* A779C polymorphism and maternal authoritarianism on creative potential, the present study also examined whether the interaction would coincide with the diathesis-stress model or the differential susceptibility model. The RoS analysis provided interesting results by showing supporting evidence for both the diathesis-stress model and the differential susceptibility model. For flexibility, it was found that the interaction was consistent with the diathesis-stress model, such that individuals with the CC genotype showed lower flexibility than those of the AA/AC genotypes when there was high maternal authoritarianism, and there was no differences when there was low maternal authoritarianism; while for originality, the differential susceptibility model best explained the interaction, in which individuals with the CC genotype showed higher originality than those of the AA/AC genotype when there was low maternal authoritarianism (i.e., “for better effects” when environment is supportive; the absence of adversities could also be considered as a type of positive environment) but lower originality than those of the AA/AC genotype when there was high maternal authoritarianism (i.e., “for worse effects” when environment is adverse). These findings suggested that the potential mechanisms underlying the G × E interaction on creativity might be complicated. For different dimensions of creativity, there might be different G × E interaction patterns, and genes could be either “risk” or “plasticity” to modulate the effect of environment on creativity.

Moreover, although three different maternal parenting styles were tested in the present study, only maternal authoritarianism was found to be interacted with *TPH1* A779C polymorphism to influence creative potential. This result was coincided with previous finding that, among the three different parenting styles, only authoritarian parenting interacted with *DRD2* to affect creative potential (Si et al., 2018). These evidences together suggested that, unlike authoritative or permissive parenting styles, the effect of authoritarian parenting on creativity might be more dependent on individuals’ genetic predispositions. For individuals with particular predispositions (e.g., the CC genotype of *TPH1* A779C polymorphism), to maximize their creative potential, avoiding the effect of adverse environment (e.g., high authoritarian parenting) might be at least as important as, or even more important than proactively creating positive environment (e.g., authoritative parenting).

Several limitations of the present study should be noted. First, the degree to which these findings could generalize to other samples is not clear. Since the participants of the present study were only Han Chinese undergraduate students, replication studies across different age and ethnic groups are necessary. Second, maternal parenting styles were only measured by self-report in the present study, thus the participants’ responses may have been subject to recall bias. Future studies combining both family observation and multi-angle measurement (e.g., mother’s report and other’s report) of maternal parenting styles are guaranteed to provide more comprehensive and convincing results. Third, although the present study provides plausible explanation for the significant interactions, the exact mechanisms underlying these interactions remains to be clarified and refined. Because serotonin is involved in multiple emotional, cognitive and behavioral control process and the modulation of serotonin transmission is a complex network of different biological process (Barnes and Sharp, 1999; Meneses, 1999; Robbins and Roberts, 2007; Cools, 2012; Jenkins et al., 2016), sorting out the nature of the interactions remains an interpretive challenge. Future studies are required to reveal both the psychological and the biological mechanisms of the interactions. For example, since serotonin has important effects on intelligence-related cognitive functions and intelligence is closely related to creativity (Silvia, 2015; Karwowski et al., 2016), it is possible that general intelligence may contribute to the interaction of *TPH1* A779C polymorphism and maternal authoritarianism on creative potential. However, since intelligence was not measured in the present study, this hypothesis could not be examined. Future studies are warranted to test this hypothesis. And it has also to be elucidated whether the effect of serotonin is directly exerted or by an indirectly interaction with the dopamine system, since it has been shown that serotonin can inhibit dopamine activation (Porras et al., 2002; Abi-Dargham, 2007). Moreover, recent genome-wide association studies (GWAS) of complex traits have shown that most complex phenotypes are highly polygenic (Manolio et al., 2009; Gratten et al., 2014; Robinson et al., 2014). As for creativity, this situation may also hold true. Since only one genetic variant (*TPH1* A779C polymorphism) was examined in the present study, the full contribution of gene and parenting styles interaction to creativity was far more from being revealed. For future studies aimed to fully uncover the interaction of gene and parenting styles on creativity, polygenic score derived from GWAS of creativity may be an ideal candidate genetic index to test the interaction of genes and parenting styles. And there has been one successful attempt using GWAS data to explore both the neural and genetic determinants of creativity (Liu et al., 2018).

In conclusion, the present study shows the first evidence for the interaction of *TPH1* A779C polymorphism and maternal authoritarianism on creative potential. These findings may provide important insight into how gene interacts with environment to influence creativity, and help to explain the origins of individual differences in creativity.

## Author Contributions

SZ and JZ were involved in the conception and design of the work. SZ, XH, and SS collected the data. SZ analyzed the data and contributed in writing the main manuscript text.

## Conflict of Interest Statement

The authors declare that the research was conducted in the absence of any commercial or financial relationships that could be construed as a potential conflict of interest.

## Footnotes

1 http://www.yourpersonality.net/interaction/
