# Supplementary material for: The Interaction of TPH1 A779C Polymorphism and Maternal Authoritarianism on Creative Potential
Source: Front Psychol. 2018 Nov 2;9:2106. doi: 10.3389/fpsyg.2018.02106 (PMC6224424; doi:10.3389/fpsyg.2018.02106)
Supplement: Supplementary file 1 [file Table_1.DOCX]

Table S1 Results of hierarchical multiple linear regression analysis testing the interaction of *TPH1* A779C polymorphism and maternal authoritarianism on fluency

|  | Fluency | | | | | | | | |
| --- | --- | --- | --- | --- | --- | --- | --- | --- | --- |
|  | Model I | | | Model II | | | Model III | | |
|  | *B (SE)* | *β* | *t* | *B (SE)* | *β* | *t* | *B (SE)* | *β* | *t* |
| *Control*  *variables* |  |  |  |  |  |  |  |  |  |
| Gender | -1.04 (.330) | -.151 | -3.13** | -1.04 (.332) | -.152 | -3.15****** | -1.03 (.331) | -.151 | -3.11****** |
| *Independent*  *variables* |  |  |  |  |  |  |  |  |  |
| M-authoritarianism | — | — | — | -.006 (.022) | -.013 | -.270 | -.010 (.022) | -.023 | -.472 |
| *TPH1* | — | — | — | .399 (.305) | .063 | 1.31 | .434 (.305) | .069 | 1.42 |
| M-authoritarianism × *TPH1* | — | — | — | — | — | — | .091 (.050) | .088 | 1.81 |
| *Model* |  |  |  |  |  |  |  |  |  |
| *R^2^* | .023 | | | .027 | | | .035 | | |
| *ΔR^2^* | .023 | | | .004 | | | .008 | | |

*Note.* **p* < .05, ***p* < .01; M-authoritarianism = maternal authoritarianism; Gender was dummy-coded as 0 = female versus 1 = male; Genotypes of *TPH1* A779C polymorphism were dummy-coded as 0 = CC versus 1 = AA/AC.

Table S2 Results of hierarchical multiple linear regression analysis testing the interaction of *TPH1* A779C polymorphism and maternal authoritativeness on creative potential

|  | Fluency | | | | | | | | |  | Flexibility | | | | | | | | |  | Originality  Model I Model II Model III  B (SE) β t B (SE) β t B (SE) b t  -.199 (.194) -.050 -1.03 -.193 (.195) -.048 -.988 -.182 (.194) -.046 -.939  — — — -.008 (.013) -.030 -.620 -.012 (.013) -.044 -.894  — — — .069 (.179) .019 .384 .096 (.179) .026 .539  — — — — — — .072 (.029) .120 2.46*  .002 .004 .018  .002 .001 .014 | | | | | | | | |
| --- | --- | --- | --- | --- | --- | --- | --- | --- | --- | --- | --- | --- | --- | --- | --- | --- | --- | --- | --- | --- | --- | --- | --- | --- | --- | --- | --- | --- | --- |
|  | Model I | | | Model II | | | Model III | | |  | Model I | | | Model II | | | Model III | | |  | Model I | | | Model II | | | Model III | | |
|  | *B (SE)* | *β* | *t* | *B (SE)* | *β* | *t* | *B (SE)* | *β* | *t* |  | *B (SE)* | *β* | *t* | *B (SE)* | *β* | *t* | *B (SE)* | *b* | *t* |  | *B (SE)* | *β* | *t* | *B (SE)* | *β* | *t* | *B (SE)* | *b* | *t* |
| *Control*  *variables* |  |  |  |  |  |  |  |  |  |  |  |  |  |  |  |  |  |  |  |  |  |  |  |  |  |  |  |  |  |
| Gender | -1.04 (.330) | -.151 | -3.13^**^ | -1.08 (.329) | -.157 | -3.27^**^ | -1.07 (.329) | -.157 | -3.25^**^ |  | -.132 (.125) | -.052 | -1.06 | -.142 (.125) | -.056 | -1.14 | -.142 (.125) | -.056 | -1.14 |  | -.199 (.194) | -.050 | -1.03 | -.216 (.193) | -.054 | -1.12 | -.219 (.194) | -.055 | -1.13 |
| *Independent*  *variables* |  |  |  |  |  |  |  |  |  |  |  |  |  |  |  |  |  |  |  |  |  |  |  |  |  |  |  |  |  |
| M-authoritativeness | — | — | — | .062 (.026) | .116 | 2.41^*^ | .062 (.026) | .116 | 2.42^*^ |  | — | — | — | .010 (.010) | .052 | 1.07 | .010 (.010) | .052 | 1.07 |  | — | — | — | .037 (.015) | .120 | 2.47^*^ | .037 (.015) | .119 | 2.45^*^ |
| *TPH1* | — | — | — | .404 (.303) | .064 | 1.34 | .403 (.303) | .064 | 1.33 |  | — | — | — | .161 (.115) | .068 | 1.40 | .161 (.115) | .068 | 1.40 |  | — | — | — | .068 (.178) | .019 | .383 | .069 (.178) | .019 | .388 |
| M-authoritativeness  ×  *TPH1* | — | — | — | — | — | — | .017 (.054) | .015 | .318 |  | — | — | — | — | — | — | .000 (.021) | .000 | -.007 |  | — | — | — | — | — | — | -.016 (.032) | -.024 | -.487 |
| *Model* |  |  |  |  |  |  |  |  |  |  |  |  |  |  |  |  |  |  |  |  |  |  |  |  |  |  |  |  |  |
| *R^2^* | .023 | | | .040 | | | .040 | | |  | .003 | | | .010 | | | .010 | | |  | .002 | | | .017 | | | .018 | | |
| *ΔR^2^* | .023 | | | .017 | | | .000 | | |  | .003 | | | .007 | | | .000 | | |  | .002 | | | .015 | | | .001 | | |

*Note.* **p* < .05, ***p* < .01; M-authoritativeness = maternal authoritativeness; Gender was dummy-coded as 0 = female versus 1 = male; Genotypes of *TPH1* A779C polymorphism were dummy-coded as 0 = CC versus 1 = AA/AC.

Table S3 Results of hierarchical multiple linear regression analysis testing the interaction of *TPH1* A779C polymorphism and maternal permissiveness on creative potential

|  | Fluency | | | | | | | | |  | Flexibility | | | | | | | | |  | Originality  Model I Model II Model III  B (SE) β t B (SE) β t B (SE) b t  -.199 (.194) -.050 -1.03 -.193 (.195) -.048 -.988 -.182 (.194) -.046 -.939  — — — -.008 (.013) -.030 -.620 -.012 (.013) -.044 -.894  — — — .069 (.179) .019 .384 .096 (.179) .026 .539  — — — — — — .072 (.029) .120 2.46*  .002 .004 .018  .002 .001 .014 | | | | | | | | |
| --- | --- | --- | --- | --- | --- | --- | --- | --- | --- | --- | --- | --- | --- | --- | --- | --- | --- | --- | --- | --- | --- | --- | --- | --- | --- | --- | --- | --- | --- |
|  | Model I | | | Model II | | | Model III | | |  | Model I | | | Model II | | | Model III | | |  | Model I | | | Model II | | | Model III | | |
|  | *B (SE)* | *β* | *t* | *B (SE)* | *β* | *t* | *B (SE)* | *β* | *t* |  | *B (SE)* | *β* | *t* | *B (SE)* | *β* | *t* | *B (SE)* | *b* | *t* |  | *B (SE)* | *β* | *t* | *B (SE)* | *β* | *t* | *B (SE)* | *b* | *t* |
| *Control*  *variables* |  |  |  |  |  |  |  |  |  |  |  |  |  |  |  |  |  |  |  |  |  |  |  |  |  |  |  |  |  |
| Gender | -1.04 (.330) | -.151 | -3.13^**^ | -1.03 (.330) | -.151 | -3.12^**^ | -1.03 (.330) | -.151 | -3.12^**^ |  | -.132 (.125) | -.052 | -1.06 | -.132 (.125) | -.052 | -1.06 | -.132 (.125) | -.051 | -1.06 |  | -.199 (.194) | -.050 | -1.03 | -.198 (.195) | -.050 | -1.02 | -.198 (.195) | -.050 | -1.02 |
| *Independent*  *variables* |  |  |  |  |  |  |  |  |  |  |  |  |  |  |  |  |  |  |  |  |  |  |  |  |  |  |  |  |  |
| M-permissiveness | — | — | — | .044 (.031) | .069 | 1.44 | .043 (.031) | .067 | 1.39 |  | — | — | — | .015 (.012) | .063 | 1.30 | .015 (.012) | .062 | 1.26 |  | — | — | — | .007 (.018) | .020 | .407 | .007 (.018) | .019 | .380 |
| *TPH1* | — | — | — | .396 (.304) | .063 | 1.30 | .394 (.304) | .062 | 1.30 |  | — | — | — | .160 (.115) | .068 | 1.40 | .159 (.115) | .068 | 1.39 |  | — | — | — | .062 (.179) | .017 | .348 | .061 (.179) | .017 | .343 |
| M-permissiveness  ×  *TPH1* | — | — | — | — | — | — | .086 (.067) | .062 | 1.28 |  | — | — | — | — | — | — | .024 (.025) | .046 | .946 |  | — | — | — | — | — | — | .027 (.040) | .033 | .675 |
| *Model* |  |  |  |  |  |  |  |  |  |  |  |  |  |  |  |  |  |  |  |  |  |  |  |  |  |  |  |  |  |
| *R^2^* | .023 | | | .032 | | | .035 | | |  | .003 | | | .011 | | | .013 | | |  | .002 | | | .003 | | | .004 | | |
| *ΔR^2^* | .023 | | | .009 | | | .004 | | |  | .003 | | | .009 | | | .002 | | |  | .002 | | | .001 | | | .001 | | |

*Note.* **p* < .05, ***p* < .01; M-permissiveness = maternal permissiveness; Gender was dummy-coded as 0 = female versus 1 = male; Genotypes of *TPH1* A779C polymorphism were dummy-coded as 0 = CC versus 1 = AA/AC.
